# Antibiotic Exposure Patterns and Clinical Outcomes Preceding Clostridioides difficile Infection: A Retrospective Observational Study

**DOI:** 10.7759/cureus.105289

**Published:** 2026-03-16

**Authors:** Kunalsen Jagatdeo, Sidharth S Pattnaik, Gyanamitra Panigrahi, Soumayan Mondal, Varun Jindal, Dipti Pattnaik, Nirmala Poddar, Shubhransu Patro

**Affiliations:** 1 Microbiology, Kalinga Institute of Medical Sciences, Bhubaneswar, IND; 2 Internal Medicine, Kalinga Institute of Medical Sciences, Bhubaneswar, IND; 3 Internal Medicine, Srirama Chandra Bhanja Medical College and Hospital, Cuttack, IND

**Keywords:** antibiotic exposure, antimicrobial stewardship program, cdi severity, clostridioides difficile infection, hospital acquired

## Abstract

Background

*Clostridioides difficile* infection (CDI) remains a major cause of healthcare-associated morbidity and mortality, with antibiotic exposure recognized as the principal modifiable risk factor. Variability in antibiotic class, duration, and overlap may influence CDI risk and clinical severity. Local audits of prescribing patterns are essential to inform antimicrobial stewardship strategies.

Objective

The objective of the study was to characterize antibiotic exposure patterns preceding laboratory-confirmed CDI and to describe associated clinical outcomes, including disease severity, recurrence, and CDI-attributable mortality in hospitalized patients.

Methods

This retrospective observational study was conducted at Kalinga Institute of Medical Sciences, Bhubaneswar, India, between January 2023 and December 2024. Adult inpatients (≥18 years) with glutamate dehydrogenase (GDH)- and toxin-positive CDI were included. Antibiotic exposure within the preceding 60 days was analyzed for class, duration, number of agents, overlap, and time to CDI. High-risk antibiotic classes were predefined. CDI severity was graded according to the Infectious Diseases Society of America (IDSA)/Society for Healthcare Epidemiology of America (SHEA) 2021 criteria. Outcomes assessed included recurrence and CDI-attributable mortality. Data were analyzed using descriptive statistics.

Results

Twenty-seven patients were included (mean age 54.26 ± 14.03 years; 59.3% male). The median cumulative antibiotic exposure was 41 days (Q1-Q3: 20.25-129), and 63.0% of patients had overlapping antibiotic therapy. The median interval from first antibiotic exposure to CDI diagnosis was 12 days (Q1-Q3: 8-30.25). Exposure to high-risk antibiotic classes ranged from zero to five, with 29.6% receiving two classes and 11.1% receiving four or more. Non-severe CDI was observed in 55.6% of patients, severe CDI in 37.0%, and fulminant CDI in 7.4%. No recurrence was documented. CDI-attributable mortality was 18.5%.

Conclusion

Prolonged and multi-class antibiotic exposure, including frequent use of high-risk agents and overlapping therapy, was common among hospitalized patients who developed CDI. The notable mortality observed underscores the importance of targeted antimicrobial stewardship interventions aimed at minimizing unnecessary broad-spectrum and concurrent antibiotic use.

## Introduction

*Clostridioides difficile* infection (CDI) is a major cause of healthcare-associated gastrointestinal illness worldwide. The global burden of CDI is substantial, with an estimated 123,997 healthcare-associated cases occurring annually in acute care hospitals across the European Economic Area and approximately 500,000 infections with nearly 29,000 deaths reported in the United States [1]. This spore-forming, Gram-positive anaerobic bacillus is responsible for a clinical spectrum ranging from mild diarrhea to severe pseudomembranous colitis, toxic megacolon, and death [1]. CDI is associated with substantial morbidity, mortality, prolonged hospitalization, and increased healthcare expenditure [2,3].

Antibiotic exposure remains the most important modifiable risk factor for both hospital-acquired and community-onset CDI [4]. Disruption of the normal intestinal microbiota following antimicrobial therapy facilitates colonization and toxin production by *C. difficile*. Although nearly all antibiotic classes have been implicated in CDI development [5], certain agents confer disproportionately higher risk. Clindamycin, later-generation cephalosporins, and fluoroquinolones are consistently categorized as high-risk antibiotics [6], whereas tetracyclines and metronidazole have traditionally been considered as lower-risk agents [7]. Given this variability in CDI risk across antibiotic classes, antimicrobial stewardship strategies increasingly emphasize risk-stratified prescribing to mitigate CDI incidence [8,9].

In addition to antibiotic exposure, multiple host- and healthcare-related factors contribute to CDI susceptibility. Advanced age, comorbid illness, recent hospitalization, intensive care unit (ICU) stay, immunosuppression, and use of gastric acid-suppressive therapy have all been associated with increased CDI risk [10,11]. Proton pump inhibitor (PPI) use in particular has been linked to both primary and recurrent CDI in observational studies [12-14]. The interplay between antimicrobial exposure, patient vulnerability, and healthcare-associated factors underscores the need for local epidemiological audits to inform stewardship interventions. Structured antimicrobial stewardship programs implemented through systematic quality-improvement approaches have demonstrated substantial reductions in antimicrobial utilization while maintaining clinical outcomes, highlighting the value of data-driven prescribing oversight in hospital settings [15]. 

Despite widespread recognition of antibiotic-associated CDI risk, real-world prescribing patterns preceding CDI in hospitalized patients remain variably characterized across institutions [16]. Evaluating the class, number, duration, and overlap of antibiotics administered prior to confirmed CDI may provide actionable insights for antimicrobial stewardship programs.

The primary objective of this study was to characterize antibiotic prescribing patterns preceding laboratory-confirmed CDI, including antibiotic class, number of agents, duration of therapy, overlap, and time from antibiotic exposure to CDI onset. The secondary objectives were to describe the frequency of CDI recurrence, CDI-attributable mortality, and severity distribution according to the Infectious Diseases Society of America/Society for Healthcare Epidemiology of America (IDSA/SHEA) 2021 criteria [17], as well as to characterize patient- and antibiotic-related factors observed among CDI cases in a tertiary care hospital setting.

## Materials and methods

Study design and setting

This retrospective observational study was conducted at Kalinga Institute of Medical Sciences (KIMS), Bhubaneswar, a tertiary care teaching hospital, to evaluate antibiotic prescribing patterns preceding laboratory-confirmed CDI. The study spanned two years, from January 2023 to December 2024.

Study population

Adult inpatients aged 18 years and above who were diagnosed with CDI during the study period were screened for eligibility. Only those patients who fulfilled the inclusion criteria and had complete clinical and antibiotic exposure records for the preceding 60 days were included in the final analysis, resulting in a study cohort of 27 patients. As this was a retrospective audit of all eligible CDI cases during the defined study period, a formal sample size calculation was not performed.

Inclusion and exclusion criteria

Patients were included if they were aged 18 years or older and had laboratory confirmation of CDI based on glutamate dehydrogenase (GDH) antigen positivity with toxin positivity on stool assay. Only those patients whose complete clinical records and antibiotic exposure history for the preceding 60 days were available were enrolled.

Patients with GDH-positive but toxin-negative results or discordant assay findings were excluded. Cases in which CDI had been diagnosed prior to the index hospitalization were also excluded. Patients with incomplete antibiotic documentation or missing clinical records were not included in the analysis.

Data collection

Data were retrieved from hospital medical records using a predesigned structured proforma. Patient identifiers were removed prior to analysis to maintain confidentiality.

Demographic variables, including age and sex, were recorded along with hospitalization details such as duration of stay, prior hospitalization, ICU admission, and recent surgical history. Clinical risk factors assessed included comorbid illnesses, immunosuppressive therapy, PPI use, and enteral feeding status. Antibiotic exposure data were obtained from hospital electronic medical records and medication charts documenting antimicrobial prescriptions during hospitalization.

CDI-related clinical and laboratory parameters

Details pertaining to CDI diagnosis included GDH and toxin assay results. Laboratory parameters, including white blood cell count, serum creatinine, and serum albumin, were documented. CDI severity was graded according to the IDSA and SHEA 2021 clinical practice guidelines. Non-severe disease was defined as a leukocyte count ≤15,000 cells/mm³ and serum creatinine <1.5 mg/dL. Severe CDI was defined as a leukocyte count >15,000 cells/mm³ or serum creatinine ≥1.5 mg/dL. Fulminant disease was defined by the presence of hypotension or shock, ileus, or toxic megacolon according to guideline criteria [17].

Definitions

CDI recurrence was defined as the reappearance of symptomatic infection within two to eight weeks following clinical resolution of the primary episode [18]. CDI-attributable mortality was defined as death occurring within 30 days of CDI diagnosis in which CDI-related complications were considered a contributing factor based on clinical documentation in the medical record.

Antibiotic exposure assessment

Antibiotic exposure within the 60 days preceding CDI diagnosis was evaluated in detail. Information regarding antibiotic name, pharmacological class, indication, route of administration, and start and end dates of therapy was recorded. Exposure variables analyzed included the total number of antibiotics prescribed, number of antibiotic cycles, cumulative duration of therapy, duration of overlapping antibiotic use, and the time interval from first antibiotic exposure to CDI onset. High-risk antibiotic classes were predefined based on existing literature and included fluoroquinolones, third- and fourth-generation cephalosporins, clindamycin, carbapenems, and β-lactam/β-lactamase inhibitor combinations [12]. For analytical purposes, antibiotic therapy preceding CDI was divided into sequential “cycles,” defined as discrete courses of antibiotic treatment separated by a change in antibiotic regimen or initiation of a new antibiotic following completion or modification of a prior course. These cycles, therefore, represent sequential treatment episodes rather than predefined phases of infection management.

Outcome measures

Outcome measures assessed in the study included the frequency and class distribution of antibiotic exposure preceding CDI, number of antibiotics prescribed, duration of therapy and overlap, severity of CDI at presentation, recurrence rates, and CDI-attributable mortality.

Ethical considerations

Institutional Ethics Committee approval was obtained with waiver of informed consent owing to the retrospective design of the study and the use of anonymized patient data.

Statistical analysis

Data were analyzed using IBM SPSS Statistics for Windows, Version 26 (Released 2018; IBM Corp., Armonk, New York, United States). Descriptive statistical methods were employed. Continuous variables were expressed as mean with standard deviation or median with interquartile range, depending on distribution. Categorical variables were summarized as frequencies and percentages. Inferential statistical testing was not performed due to the small sample size, which limited the statistical power for association analyses and regression modeling.

## Results

A total of 27 adult inpatients with laboratory-confirmed CDI were included in the final analysis.

Baseline characteristics

The mean age of the study population was 54.26 ± 14.03 years, with a male predominance (16 male patients, 11 female patients). The most common comorbidities were diabetes mellitus (12, 44.4%) and hypertension (8, 29.6%), followed by chronic kidney disease (6, 22.2%) and malignancy (4, 14.8%). Inflammatory bowel disease and hypothyroidism were present in four (14.8%) and three (11.1%) patients, respectively. Detailed baseline characteristics are presented in Table 1.

**Table 1:** Baseline Characteristics of the Study Population (N = 27) ICU: intensive care unit, WBC: white blood cell, SD: standard deviation, Q1-Q3: interquartile range

Parameter	Value
Age (years), mean ± SD	54.26 ± 14.03
Sex (Male:Female)	16:11
Comorbidities, n (%)	
Diabetes mellitus	12 (44.4%)
Hypertension	8 (29.6%)
Chronic kidney disease	6 (22.2%)
Malignancy	4 (14.8%)
Inflammatory bowel disease	4 (14.8%)
Hypothyroidism	3 (11.1%)
Others	4 (14.8%)
Hospitalization and risk factors, n (%)	
Prior hospitalization	15 (55.6%)
ICU stay	16 (59.3%)
Recent surgery	6 (22.2%)
Immunosuppressive therapy	6 (22.2%)
Enteral feeding	27 (100%)
Proton pump inhibitor use	27 (100%)
Laboratory parameters	
WBC count (cells/mm³), median (Q1-Q3)	10,770 (2,890-17,300)
Serum creatinine (mg/dL), median (Q1-Q3)	0.76 (0.50-1.02)
Serum albumin (g/dL), mean ± SD	2.6 ± 0.56

With respect to hospitalization history, 15 patients (55.6%) had a prior hospitalization within the recent period. ICU stay was documented in 16 patients (59.3%), and 6 patients (22.2%) had undergone recent surgery. Immunosuppressive therapy was administered in 6 cases (22.2%). All patients (27, 100%) were receiving PPIs and enteral feeding at the time preceding CDI diagnosis.

The median white blood cell count was 10,770 cells/mm³ (Q1-Q3: 2,890-17,300), and the median serum creatinine was 0.76 mg/dL (Q1-Q3: 0.50-1.02). The mean serum albumin level was 2.6 ± 0.56 g/dL, indicating a high prevalence of hypoalbuminemia in the cohort.

CDI severity and clinical outcomes

**Figure 1:**
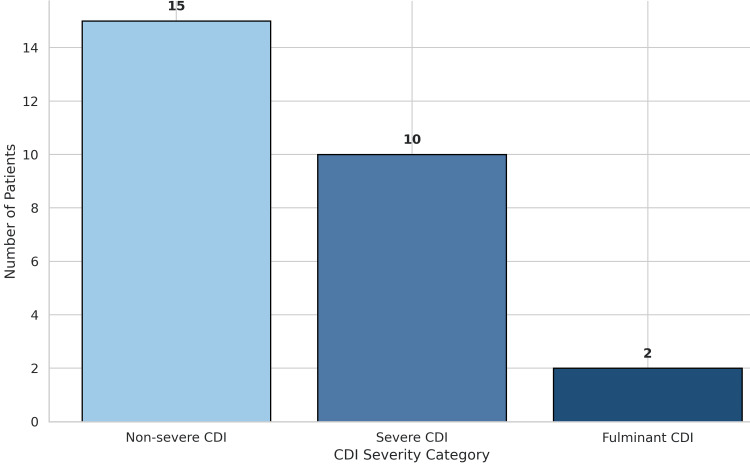
Severity Distribution of Clostridioides difficile Infection in the Study Population Bar graph depicting the distribution of disease severity among hospitalized patients diagnosed with *Clostridioides difficile* infection. Severity classification was performed according to the Infectious Diseases Society of America/Society for Healthcare Epidemiology of America (IDSA/SHEA) 2021 clinical practice guidelines. Non-severe cases constituted the largest proportion, followed by severe and fulminant disease categories. CDI: Clostridioides difficile infection, IDSA: Infectious Diseases Society of America, SHEA: Society for Healthcare Epidemiology of America

CDI severity was graded according to IDSA/SHEA 2021 criteria. Fifteen patients (55.6%) were classified as having non-severe disease, ten patients (37.0%) had severe CDI, and two patients (7.4%) were diagnosed with fulminant disease. The distribution of severity categories is shown in Figure 1.

No cases of CDI recurrence were observed during the defined follow-up period. CDI-attributable mortality occurred in 5 patients, corresponding to a mortality rate of 18.5%.

Antibiotic exposure patterns

Antibiotic exposure within the 60 days preceding CDI diagnosis varied across patients. Three patients (11.1%) had no documented antibiotic exposure, whereas the remaining 24 patients (88.9%) received at least one course of antibiotics. Multiple antibiotic exposure was common, with several patients receiving three or more agents during the observation period.

The median cumulative duration of antibiotic therapy was 41 days (Q1-Q3: 20.25-129 days). Antibiotic overlap, defined as concurrent administration of two or more agents, was observed in 17 patients (63.0%), with a median overlap duration of 11 days (Q1-Q3: 5.5-15.5 days). The median interval from initiation of the first antibiotic to CDI diagnosis was 12 days (Q1-Q3: 8-30.25 days). These exposure metrics are summarized in Table 2.

**Table 2:** Overall Antibiotic Exposure Characteristics Prior to CDI CDI: Clostridioides difficile infection, Q1-Q3: interquartile range

Parameter	Median	Q1-Q3
Total antibiotic exposure (days)	41	20.25-129.00
Antibiotic overlap duration (days)	11	5.50-15.50
Time from first antibiotic to CDI (days)	12	8.00-30.25

Across five potential antibiotic cycles, ceftriaxone was the most frequently prescribed agent during the first cycle, followed by piperacillin-tazobactam and meropenem in subsequent cycles. Later cycles more commonly included metronidazole and teicoplanin. Duration of therapy across cycles ranged from a median of 5.5 to 13.5 days. Detailed cycle-wise prescribing characteristics are shown in Table 3.

**Table 3:** Cycle-Wise Antibiotic Prescribing Characteristics High-risk antibiotics include fluoroquinolones, third- and fourth-generation cephalosporins, clindamycin, carbapenems, and β-lactam/β-lactamase inhibitor combinations. Q1-Q3: interquartile range

Parameter	Cycle 1	Cycle 2	Cycle 3	Cycle 4	Cycle 5
Duration of therapy (days), median (Q1–Q3)	10.5 (5-38)	9 (3.25-17.75)	13.5 (4.75-91)	7 (4-13.75)	5.5 (1.75-33)
Most prescribed antibiotic	Ceftriaxone	Piperacillin–tazobactam	Meropenem	Metronidazole	Teicoplanin
High-risk antibiotic use, n (%)	18 (66.7%)	13 (48.1%)	12 (44.4%)	4 (14.8%)	3 (11.1%)
Intravenous route, n (%)	20 (74.1%)	17 (63.0%)	13 (48.1%)	7 (25.9%)	5 (18.5%)
Oral route, n (%)	4 (14.8%)	3 (11.1%)	5 (18.5%)	5 (18.5%)	1 (3.7%)

**Figure 2:**
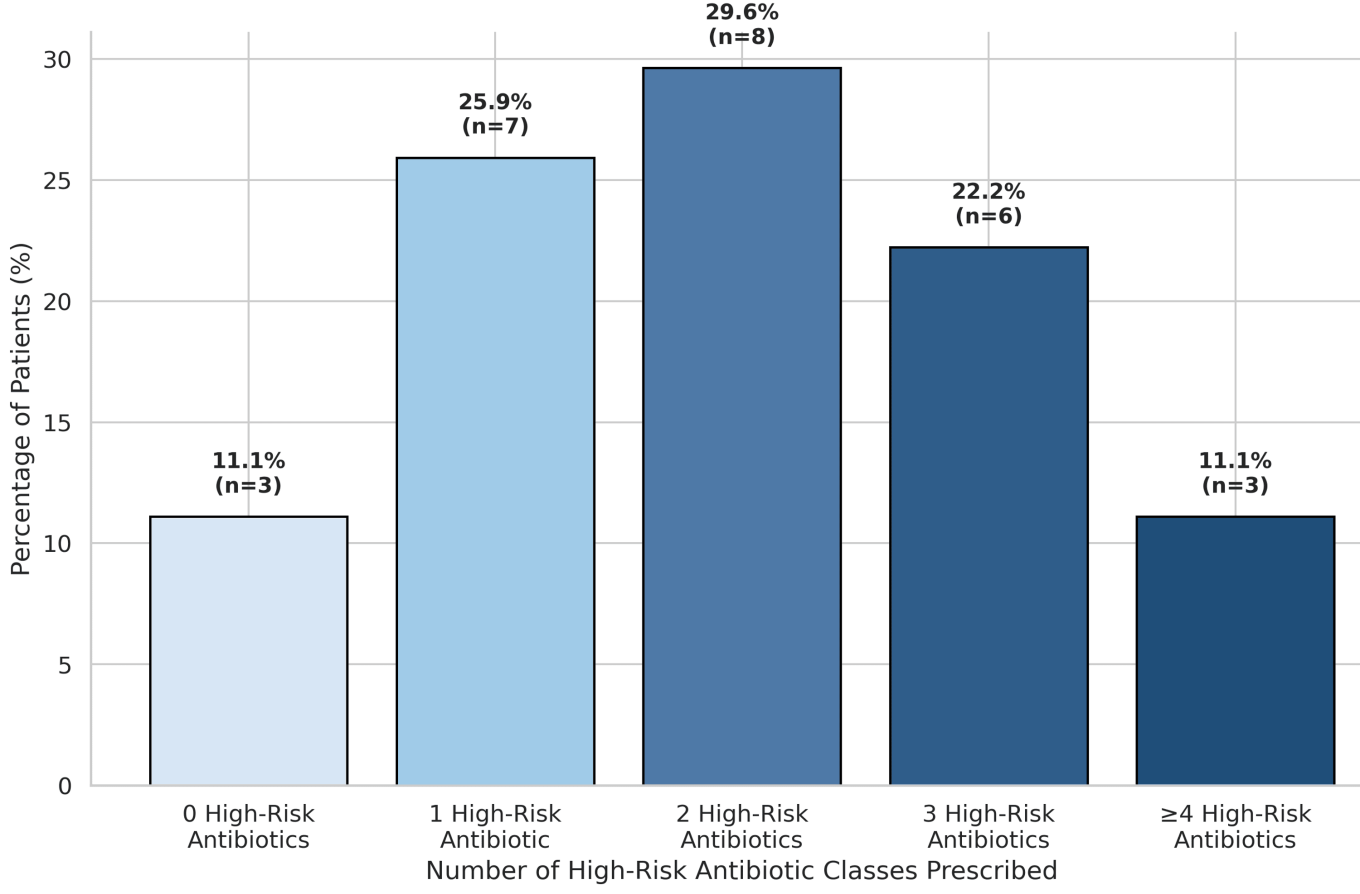
Distribution of High-Risk Antibiotic Class Exposure Preceding CDI Bar graph illustrating the distribution of patients according to the number of high-risk antibiotic classes prescribed within the 60 days preceding *Clostridioides difficile* infection diagnosis. Exposure ranged from zero to five high-risk antibiotic classes; categories with four and five classes were combined due to small sample size. High-risk antibiotics were defined as fluoroquinolones, third- and fourth-generation cephalosporins, clindamycin, carbapenems, and β-lactam/β-lactamase inhibitor combinations. CDI: Clostridioides difficile infection

High-risk antibiotic classes, including third- and fourth-generation cephalosporins, fluoroquinolones, carbapenems, clindamycin, and β-lactam/β-lactamase inhibitor combinations, were commonly prescribed. The frequency distribution of high-risk antibiotic exposure across cycles is depicted in Figure 2. A substantial proportion of patients received more than one high-risk antibiotic during the exposure window.

Intravenous administration predominated during earlier antibiotic cycles, particularly cycle 1 (74.1%) and cycle 2 (63.0%), with a gradual reduction in IV use in later cycles. Oral antibiotic use was less frequent overall.

## Discussion

This retrospective observational study evaluated antibiotic prescribing patterns preceding CDI and examined associated host and treatment-related risk factors in hospitalized patients. The findings reinforce the central role of antimicrobial exposure, particularly prolonged duration, overlapping therapy, and multi-class prescribing, in CDI pathogenesis while also highlighting contributory clinical vulnerabilities such as gastric acid suppression and hypoalbuminemia.

Antibiotic exposure remains the most important modifiable risk factor for CDI. In the present cohort, most patients had received antecedent antimicrobial therapy, with a median cumulative exposure of 41 days. Prior literature demonstrates that CDI risk increases substantially with longer antibiotic courses, reflecting sustained disruption of the intestinal microbiome [19]. Moreover, the risk remains elevated for several months following antibiotic cessation due to delayed recovery of protective gut flora [19]. These findings are consistent with the prolonged exposure observed in our study.

Beyond duration, concurrent administration of multiple antibiotics represents an additional microbiological insult. Antibiotic overlap was documented in a substantial proportion of patients. Simultaneous exposure to multiple agents has been shown to amplify microbiota depletion and facilitate *C. difficile *colonization [6]. In addition, exposure to multiple high-risk antibiotic classes was frequent, with some patients receiving four or more such agents. Cumulative antibiotic burden has previously demonstrated a dose-response association with CDI risk, further supporting the role of multi-class exposure as an important determinant of infection [20].

With respect to antibiotic class, ceftriaxone, piperacillin-tazobactam, meropenem, metronidazole, and teicoplanin were among the most frequently prescribed antecedent agents. Third-generation cephalosporins and carbapenems are well recognized as high-risk antibiotics due to their broad-spectrum activity and profound impact on gut anaerobic flora [12,21]. Beta-lactam and beta-lactamase inhibitor combinations similarly increase CDI risk by enhancing suppression of commensal anaerobic organisms such as Bacteroides species [22]. Epidemiologic data further support this risk stratification. A longitudinal case-cohort analysis demonstrated markedly elevated CDI risk with cephalosporins, clindamycin, and fluoroquinolones [16]. Our findings, therefore, align with established antibiotic risk hierarchies.

An unexpected observation was the frequent antecedent use of metronidazole. Historically considered a lower-risk antibiotic and previously recommended as first-line therapy for mild CDI, metronidazole has not been strongly associated with CDI acquisition [23]. Its prominence in this cohort should therefore be interpreted cautiously. Possible explanations include confounding by indication, prior treatment of anaerobic or intra-abdominal infections, or sequential antibiotic exposure preceding CDI diagnosis. These findings should be considered hypothesis-generating rather than causative.

Host-related factors also contributed to CDI susceptibility. PPI exposure was universal in this cohort. Gastric acid suppression has been repeatedly associated with increased CDI risk, although the mechanism extends beyond simple acid barrier loss because *C. difficile* spores are acid-resistant [12]. A meta-analysis demonstrated a significant association between PPI use and CDI acquisition [13], while subsequent work has linked acid suppression to recurrent infection risk [14]. However, the universal exposure observed in this study may also reflect institutional prescribing practices, including routine stress ulcer prophylaxis in critically ill patients, and should therefore be interpreted cautiously.

Hypoalbuminemia was another prominent finding. Low serum albumin has been consistently associated with severe and complicated CDI as well as increased mortality. Inflammatory states promote capillary leak and redistribution of albumin into the interstitial space [24], while hepatic synthesis may be impaired in critical illness [25]. Protein-losing enteropathy described in CDI further contributes to hypoalbuminemia [26]. These mechanisms suggest that albumin may function both as a marker of disease severity and as a mediator of adverse outcomes.

Severity assessment demonstrated that while most patients had non-severe disease, a substantial proportion developed severe or fulminant CDI. Severe CDI is associated with complications such as toxic megacolon, perforation, and organ failure [17]. The observed CDI-attributable mortality rate of 18.5% falls within ranges reported in multicenter cohorts, where mortality has varied between approximately 10% and 30% depending on disease severity and comorbidity burden [1]. This highlights the continued clinical impact of CDI despite advances in infection control and therapeutics.

No recurrence events were documented in this study. This contrasts with published recurrence rates of 10-30% following primary infection [18]. The absence of recurrence in our cohort may reflect the limited sample size, shorter follow-up duration, or early mortality in severe cases, precluding recurrence assessment. Larger longitudinal studies would be required to more accurately evaluate recurrence risk.

Additional risk modifiers identified in prior literature, including malignancy, immunosuppressive therapy, enteral feeding, and recent surgery, were also present within the study population [27,28]. These factors likely interact synergistically with antibiotic exposure to facilitate CDI development in hospitalized patients.

This study has several limitations. The retrospective design limits causal inference, and the relatively small sample size restricted statistical power for inferential analysis and regression modeling. The absence of a control group prevented comparative risk estimation. Microbiological strain typing and antimicrobial resistance profiling were not available. Institutional prescribing practices may further limit generalizability to other healthcare settings.

The findings underscore the importance of antimicrobial stewardship interventions targeting not only antibiotic selection but also duration, overlap, and cumulative exposure burden. Restrictive prescribing strategies and de-escalation protocols have previously demonstrated reductions in CDI incidence and associated mortality [9]. Surveillance of high-risk antibiotic utilization may therefore represent a practical strategy for CDI prevention in hospitalized populations. Future preventive strategies may also include vaccine-based and immunotherapeutic approaches targeting *C. difficile* toxins, which are increasingly being investigated as complementary tools to antimicrobial stewardship for reducing infection burden and antimicrobial resistance [29].

## Conclusions

Prolonged and multi-class antibiotic exposure, including frequent use of high-risk agents and overlapping therapy, was common among hospitalized patients who developed CDI. Universal PPI use and prevalent hypoalbuminemia reflected additional vulnerability within the cohort. Although most cases were non-severe, the observed mortality underscores the clinical impact of CDI. These findings support strengthened antimicrobial stewardship efforts focused on optimizing antibiotic selection, duration, and combination use to reduce CDI risk.
